# Molecules that Generate Fingerprints: A New Class of Fluorescent Sensors for Chemical Biology, Medical Diagnosis, and Cryptography

**DOI:** 10.1021/acs.accounts.3c00162

**Published:** 2023-06-19

**Authors:** Leila Motiei, David Margulies

**Affiliations:** Department of Chemical and Structural Biology, Weizmann Institute of Science, Rehovot 7610001, Israel

## Abstract

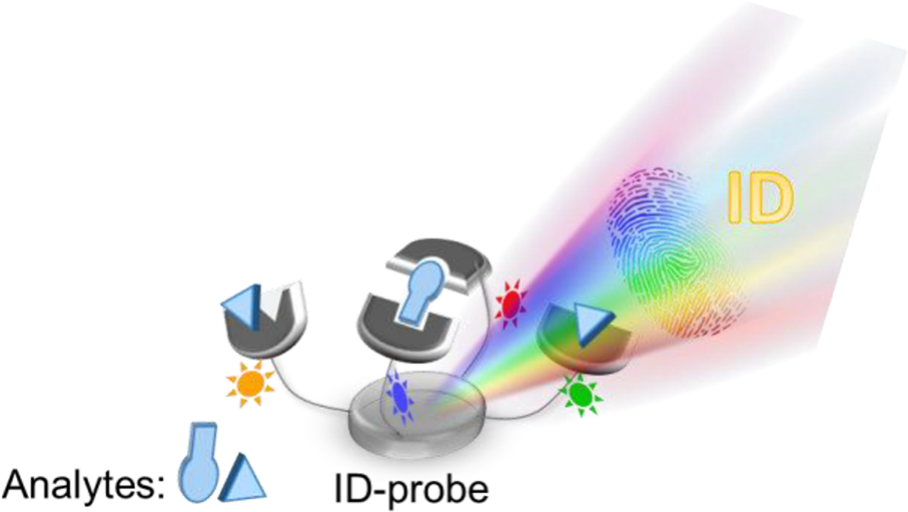

Fluorescent molecular sensors, often referred to as “turn-on” or “turn-off” fluorescent probes, are synthetic agents that change their fluorescence signal in response to analyte binding. Although these sensors have become powerful analytical tools in a wide range of research fields, they are generally limited to detecting only one or a few analytes. Pattern-generating fluorescent probes, which can generate unique identification (ID) fingerprints for different analytes, have recently emerged as a new class of luminescent sensors that can address this limitation. A unique characteristic of these probes, termed ID-probes, is that they integrate the qualities of conventional small-molecule-based fluorescent sensors and cross-reactive sensor arrays (often referred to as chemical, optical, or electronic noses/tongues). On the one hand, ID-probes can discriminate between various analytes and their combinations, akin to array-based analytical devices. On the other hand, their minute size enables them to analyze small-volume samples, track dynamic changes in a single solution, and operate in the microscopic world, which the macroscopic arrays cannot access.

Here, we describe the principles underlying the ID-probe technology, as well as provide an overview of different ID-probes that have been developed to date and the ways they can be applied to a wide range of research fields. We describe, for example, ID-probes that can identify combinations of protein biomarkers in biofluids and in living cells, screen for several protein inhibitors simultaneously, analyze the content of Aβ aggregates, as well as ensure the quality of small-molecule and biological drugs. These examples highlight the relevance of this technology to medical diagnosis, bioassay development, cell and chemical biology, and pharmaceutical quality assurance, among others. ID-probes that can authorize users and protect secret data are also presented and the mechanisms that enable them to hide (steganography), encrypt (cryptography), and prevent access to (password protection) information are discussed.

The versatility of this technology is further demonstrated by describing two types of probes: unimolecular ID-probes and self-assembled ID-probes. Probes from the first type can operate inside living cells, be recycled, and their initial patterns can be more easily obtained in a reproducible manner. The second type of probes can be readily modified and optimized, allowing one to prepare various different probes from a much wider range of fluorescent reporters and supramolecular recognition elements. Taken together, these developments indicate that the ID-probe sensing methodology is generally applicable, and that such probes can better characterize analyte mixtures or process chemically encoded information than can the conventional fluorescent molecular sensors. We therefore hope that this review will inspire the development of new types of pattern-generating probes, which would extend the fluorescence molecular toolbox currently used in the analytical sciences.

## Key References

- RoutB.; UngerL.; ArmonyG.; IronM. A.; MarguliesD.Medication Detection by a Combinatorial Fluorescent Molecular Sensor. Angew. Chem. Int. Ed.2012, 51, 12477–12481.10.1002/anie.20120637423065749(1) Describes the development of the first ID-probe which can discriminate among medications.
- SarkarT.; SelvakumarK.; MotieiL.; MarguliesD.Message in a Molecule. Nat. Commun.2016, 7, 11374.2713846510.1038/ncomms11374PMC4857388(2) Shows an ID-probe that can encrypt secret messages by operating as a molecule-size Enigma machine.
- PodeZ.; Peri-NaorR.; GeorgesonJ. M.; IlaniT.; KissV.; UngerT.; MarkusB.; BarrH. M.; MotieiL.; MarguliesD.Protein Recognition by a Pattern-Generating Fluorescent Molecular Prob*e*. Nat. Nanotechnol.2017, 12, 1161–1168.2903540010.1038/nnano.2017.175(3) With this ID-probe, which can differentiate between combination of protein isoforms in complex mixtures, the ability to apply artificial “nose/tongue” devices in confined microscopic environment such a within living cells, was demonstrated.
- Peri-NaorR.; PodeZ.; Lahav-MankovskiN.; RabinkovA.; MotieiL.; MarguliesD.Glycoform Differentiation by a Targeted, Self-Assembled, Pattern-Generating Protein Surface Sensor. J. Am. Chem. Soc.2020, 142, 15790–15798.3278675510.1021/jacs.0c05644(4) Highlights the simplicity by which complex ID-probe structures could be created from oligonucleotide building blocks. With this approach self-assembled ID-probes, which can straightforwardly differentiate between the glycosylation states of a therapeutic glycoprotein, were generated.

## 1 Introduction

**Figure 1:**
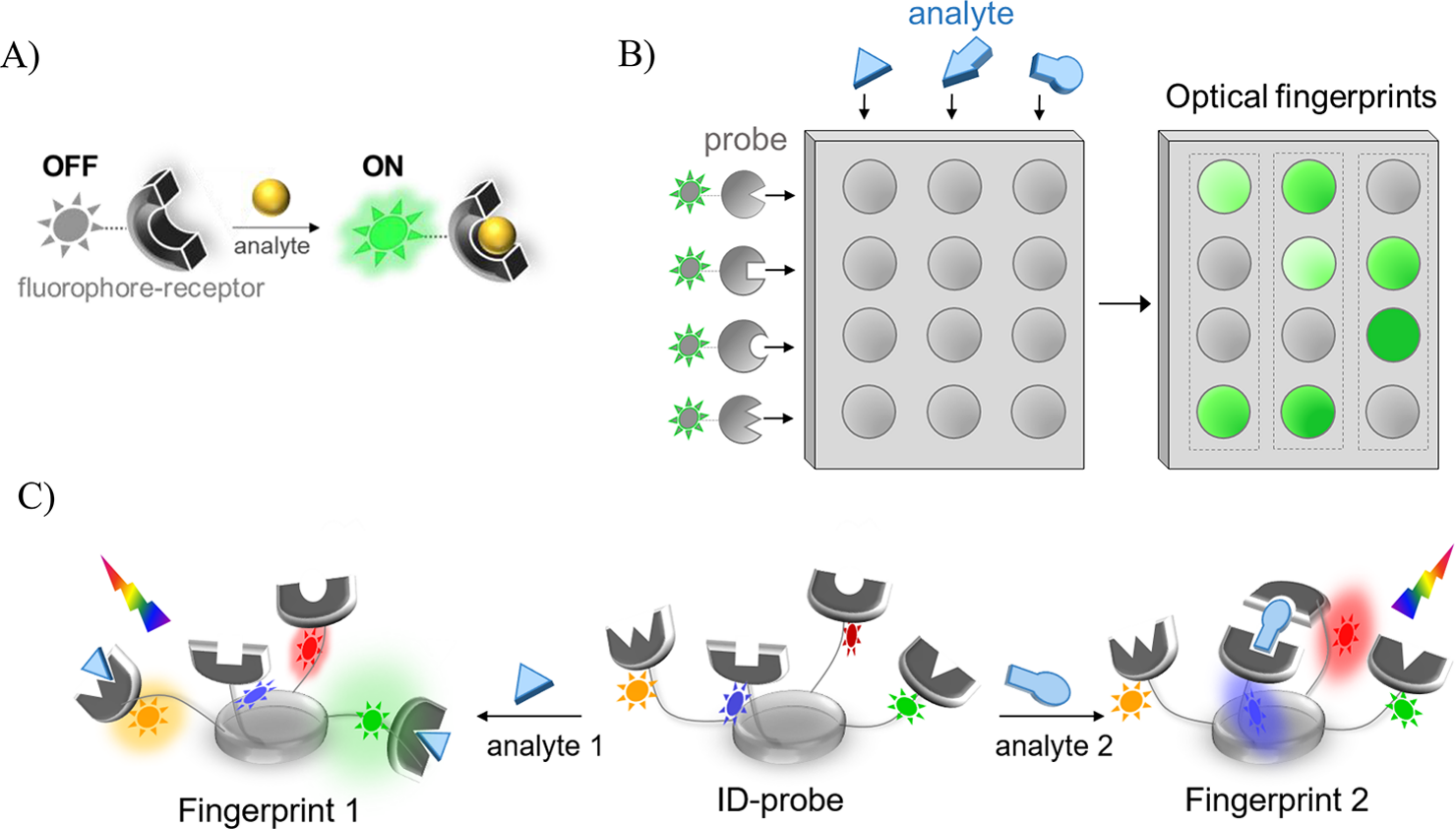
(A) Detection of a specific analyte by a “turn-on” fluorescent probe. (B) Pattern-based recognition of multiple analytes by a cross-reactive sensor array. (C) Operating principle of an ID-probe.

Fluorescent molecular sensors are synthetic agents that, upon binding to target analytes, undergo changes in their physical or chemical properties, subsequently altering their fluorescence signal (Figure 1A). Owing to their high sensitivity, diversity, small scale, and ability to provide rapid detection in distinct environments, these sensors have been broadly used in numerous fields including medical diagnosis, cell and chemical biology, pharmacology, and environmental sciences.^5−8^ These sensors, often termed “turn-on” or “turn-off” fluorescent probes, are normally constructed by conjugating a synthetic receptor to a fluorescent dye (Figure 1A). Owing to the proximity between the two, the binding of an analyte to the receptor can interfere with photophysical processes, such as photoinduced electron transfer (PET), intramolecular charge transfer (ICT), or excimer emission.^9,10^ The analyte-induced perturbation of these processes is responsible for changes in the emission intensity of such sensors. Efficient sensing often requires that the receptor will bind to its analyte with high affinity and selectivity, and that the dyes will generate a strong, turn-on fluorescence response. A large enhancement in emission intensity is important to minimize the background fluorescence generated by unbound probes, whereas potent binding ensures that low concentrations of analytes will be selectively recognized. Analogously to nature, these sensors recognize their analytes according to the “lock-and-key” mechanism^11^ underlying the strong and selective interactions between proteins and their targets, for example, enzyme–substrate, antibody–antigen, or receptor–ligand interactions.

The olfactory receptors (ORs) are an exceptional group of proteins that do not bind to their targets according to the “lock-and-key” principle. Unlike enzymes, antibodies, or most receptor proteins, which normally interact with one target, each OR can nonselectively interact with a wide range of volatile compounds.^12^ In this way, numerous odorant molecules can be distinguished according to the unique neural response pattern generated for each odorant. Understanding the way in which the olfactory system operates has inspired the development of a wide range of analytical devices that imitate it,^11,13−19^ namely, devices based on arrays of probes that are nonspecific (or cross-reactive); hence, they can generate distinct identification patterns for different analytes. Recently, these cross-reactive sensor arrays, also termed differential sensors^14,20^ (as well as chemical, optical, or electronic noses/tongues) have been applied to differentiate between biomolecules in aqueous solutions or between cells. With such systems, the biomolecule analytes can be distinguished according to the optical (generally fluorescence) fingerprints, generated by the nonspecific probes in an array (Figure 1B).^20−29^

Figure 1A and B thus not only illustrate two distinct analytical systems—they also highlights two fundamentally different approaches used to sense analytes with fluorescent molecular probes. The first approach utilizes the lock-and-key principle, whereas the second approach relies on differential sensing (or the “nose/tongue” approach). Sensors that belong to the first class (Figure 1A) are commonly applied to detect a single analyte with high affinity and selectivity, even in complex mixtures. In contrast, the second type of sensors (Figure 1B) is used to differentiate between multiple different analytes and their combinations. The relevance of differential sensors to various research fields such as medical diagnosis and proteomics has been demonstrated with various arrays that can discriminate between a wide range of medicinally relevant samples such as those containing proteins, cells, and biofluids.^20−29^

A few years ago, another fundamental difference between the two sensory systems attracted our attention. We noted that sensors that belong to the first class (Figure 1A) are generally based on a single, unimolecular fluorescent probe.^5−10^ In contrast, most differential sensors (Figure 1B) are macroscopic devices or multicomponent systems consisting of spatially separated probes.^20−31^ Although this difference is rather obvious, we realized that the small scale and homogeneity of the unimolecular sensors make them more suitable for a wide range of applications, regardless of the mechanism by which they operate. For example, only small-molecule-based probes can operate within living cells, where macroscopic arrays cannot access. In addition, they are very efficient in following dynamic changes that occur in a single solution. Furthermore, the unimolecular sensors can serve as molecule-size computing devices that process information at the molecular scale.^32^ Therefore, it occurred to us that many of the limitations associated with the use of cross-reactive sensor arrays could be circumvented if their size could be reduced to the level of an individual fluorescent molecule (Figure 1C).^1,33^ The idea is rather simple. Instead of using an array to integrate various nonspecific fluorescent probes (Figure 1B), all of them could be assembled on a single molecular platform (Figure 1C). In addition, in order to be able to read the fluorescence response of probes that are not spatially separated, each of them should emit at a different wavelength. In this way, the effect of analyte binding on the fluorescence of each dye could be detected and the different analytes could be distinguished according to the unique emission fingerprints generated by a molecule-size differential sensor. Because such unimolecular, pattern-generating probes can generate a unique identification (ID) pattern for each analyte, they were recently termed ID-probes.^3^

Following the development of the first ID-probe,^1^ we anticipated that this sensor class could offer various potential advantages.^33^ For example, we envisioned that with ID-probes, differential sensing could be applied in the microscopic world, where macroscopic “noses/tongues” cannot enter. Since then, we have proven this hypothesis by developing a pattern-generating probe that can operate within living cells and can straightforwardly discriminate between intracellular states.^3^ By developing distinct types of ID-probes,^1−4,34−36,41^ we have further shown the relevance of the ID-probe technology to a wide range of applications. Herein we describe the design and function of the ID-probes developed to date, summarize their unique properties, and provide an updated perspective on the ways these systems could be further improved and utilized.

## 2 Design and Operating Principles of ID-Probes

Our methodology to create ID-probes is to integrate a wide range of fluorescent dyes, including optically responsive dyes, and nonspecific (or partially specific) synthetic receptors on one molecular scaffold (Figure 1C). Binding of an analyte to a receptor can directly affect the fluorescence of an adjacent dye, akin to the mechanism underlying the conventional fluorescent sensors (Figure 1A). In addition, changes that occur in the emission of one dye can affect the emission of the other dyes, owing to intramolecular FRET processes. The combined effects afford the unique emission fingerprints that the ID-probes generate. As is done with cross-reactive sensor arrays, the optical fingerprints can be differentiated by using dimensionality reduction algorithms, such as principal component analysis (PCA) or linear discriminant analysis (LDA).^37^ One question that may rise regarding this design is whether the integration of various fluorescent dyes within an individual ID-probe (Figure 1C) is really necessary. One can argue that emission fingerprints could also be generated from probes bearing a single dye type. For instance, dyes that form excimers^9,10^ can produce patterns consisting of the monomer and excimer emissions. Similarly, individual lanthanide complexes can produce several emission peaks. We believe that for many applications, combining distinct types of fluorescence reporters within a single ID-probe would improve the discrimination efficiency. The combination of dyes not only increases the color variability of the system—it also diversifies the types of photophysical processes that are affected by analyte binding. This diversification increases the chances to obtain opposite fluorescence responses from the same dye and consequently, increases the number of distinguishable patterns that an individual ID-probe can produce.

## 3 Unimolecular ID-Probes

The term unimolecular ID-probe refers to pattern-generating sensors in which the various recognition elements and fluorescent reporters are covalently attached. Since the inception of the first ID-probe,^1^ various unimolecular analytical systems of this class have been developed and used for different applications.

### 3.1 High-Throughput Identification of Medications by the First ID-Probe

**Figure 2:**
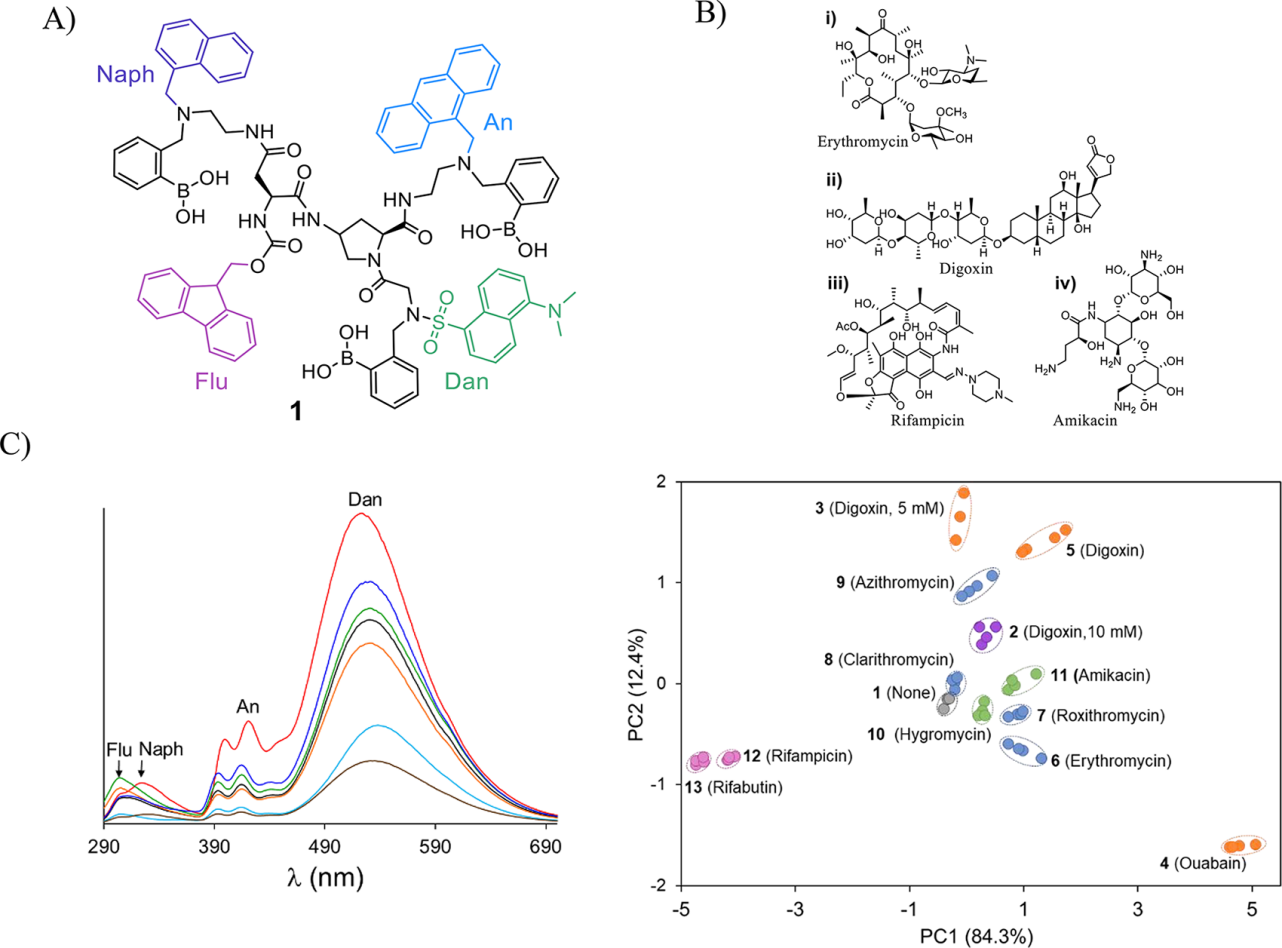
(A) Structure of ID-probe **1**. (B) Representative structures of the (i) macrolide, (ii) cardiacglycoside, (iii) rifamycin, and (iv) aminoglycoside drug families. (C) Representative emission signatures generated by **1** in response to these drugs (left), and the PCA mapping of these emission patterns (right). Adapted with permission from ref (1). Copyright 2012 John Wiley & Sons, Inc.

The first ID-probe^1^ (Figure 2A, probe **1**) was designed to discriminate between different carbohydrates and carbohydrate-based medications (Figure 2B) associated with counterfeiting or medication errors. The structure of **1** consists of a *cis*-amino proline scaffold appended with three phenyl boronic acids (BAs) and four dyes, namely, naphthalene (Naph), fluorenyl (Flu), anthracene (An), and dansyl (Dan). The BAs form the diol-binding receptors, whereas the dyes serve as fluorescent reporters. These dyes were selected owing to their partially overlapping absorption and emission spectra, affording intramolecular FRET communication. In addition, Dan is a solvatochromic probe, whereas the An-BA conjugates have been shown to act as carbohydrate sensors that are activated via several potential mechanisms.^10^ Incubating **1** with different medications resulted in markedly distinct fluorescence signatures (Figure 2C, left) that could be readily differentiated (Figure 2C, right), indicating the relevance of the ID-probe technology to pharmaceutical quality assurance. The ability of **1** to identify different combinations of rifampicin and D-xylose levels in urine^1^ showed the potential for using ID-probes in pharmacokinetics and therapeutic drug monitoring. The exceptional analytical capabilities of **1** were further demonstrated by its ability to discriminate between structurally similar saccharides, including D- and L-saccharides.^36^

### 3.2 Analyzing Dynamic Changes in the Composition of Amyloid Beta Aggregates or Synthetic Self-Replicators

**Figure 3:**
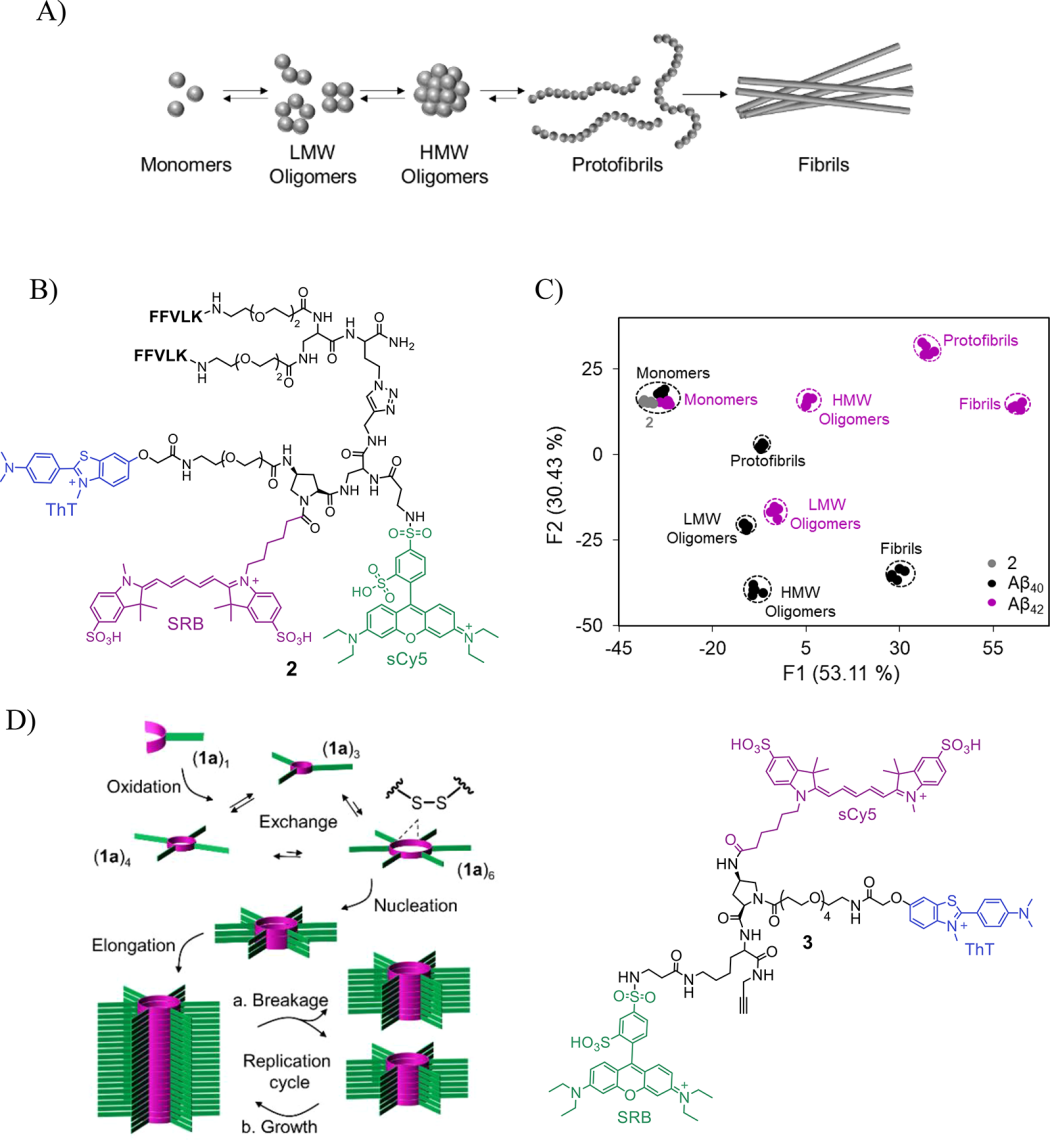
(A) Illustration of the Aβ aggregation process. (B) Structure of ID-probe **2**. (C) LDA mapping of the patterns generated by **2** in response to different Aβ aggregate species. Adapted from ref (35). Copyright 2017 American Chemical Society. (D) High-throughput analysis of self-replication (left) was achieved using ID-probe **3** (right). Adapted from ref (41). Copyright 2022 American Chemical Society.

Fluorescent molecular sensors are used in a wide range of biologically relevant assays.^38^ For example, a common assay used to follow the formation of amyloid beta (Aβ) aggregates is based on the “turn-on” response of thioflavin T (ThT) to Aβ fibrils. Although fibril formation has been associated with Alzheimer’s disease (AD) progression, various studies indicate that intermediate Aβ aggregate species, such as low molecular weight (LMW) oligomers (Figure 3A), more significantly contribute to neurotoxicity. Because these intermediates cannot be detected by ThT, analyzing the composition of Aβ aggregates relies on more complex techniques, such as mass spectrometry, gel electrophoresis, or immunoblotting, which require special expertise and are not high throughput. Cross-reactive sensor arrays (Figure 1B) can be used to differentiate between mixtures. However, the need to channel each analyte sample to different wells in an array makes such systems unsuitable for detecting changes in the Aβ aggregate composition. In particular, it leads to a high consumption of samples and decreases the detection accuracy owing to the difficulty to reproduce dynamic Aβ aggregation processes in multiple wells. These drawbacks have inspired us to develop ID-probe **2** (Figure 3B), whose fluorescence fingerprints reflect different Aβ aggregation states (Figure 3C).^35^

The structure of **2** consists of an amino proline scaffold appended with three fluorescent reporters: ThT, sulforhodamine B (SRB), and sulfo-Cy5 (sCy5) that serve as a FRET donor, acceptor/donor, and acceptor, respectively. In addition, it consists of two amyloid binders: a bis-KLVFF peptide, which enables it to bind LMW oligomers, and ThT, which can interact with fibrils, protofibrils, and high molecular weight (HMW) oligomers. We have shown that changes that occur in Aβ aggregation states can be straightforwardly differentiated, in a single solution, according to the unique optical fingerprints generated by **2** (Figure 3C). In addition, **2** was used to discriminate between aggregates generated from different alloforms, through distinct pathways or from distinct amyloidogenic proteins.

Synthetic self-replicating systems have emerged as a powerful tool for studying the synthesis and origin of life.^39^ However, the monitoring of these systems has largely relied on techniques such as NMR or chromatography, which are not high-throughput and are unsuitable for real-time monitoring of the replication process when using small volume samples with low replicator concentrations. Similar to Aβ proteins, self-replicators based on disulfide macrocycles form distinct aggregate species that self-assemble into β sheet fibrils (Figure 3D, left).^40^ Moreover, these fibrils can induce the turn-on response of ThT. This resemblance between Aβ aggregation and self-replicating process properties stimulated the development of ID-probe **3** (Figure 3D, right).^41^ In this study, which is one of the first to integrate principles from systems chemistry^39^ and differential sensing, **3** was applied to differentiate between replicators of a slightly different chemical nature and to monitor replicator formation in real time. Based on previous success in operating ID-probes in living cells,^3^ it was suggested that this study could open the way to investigate self-replication in protocell environments.^41^

### 3.3 Analyzing Protein Subpopulations in Biofluids and in Living Cells

**Figure 4:**
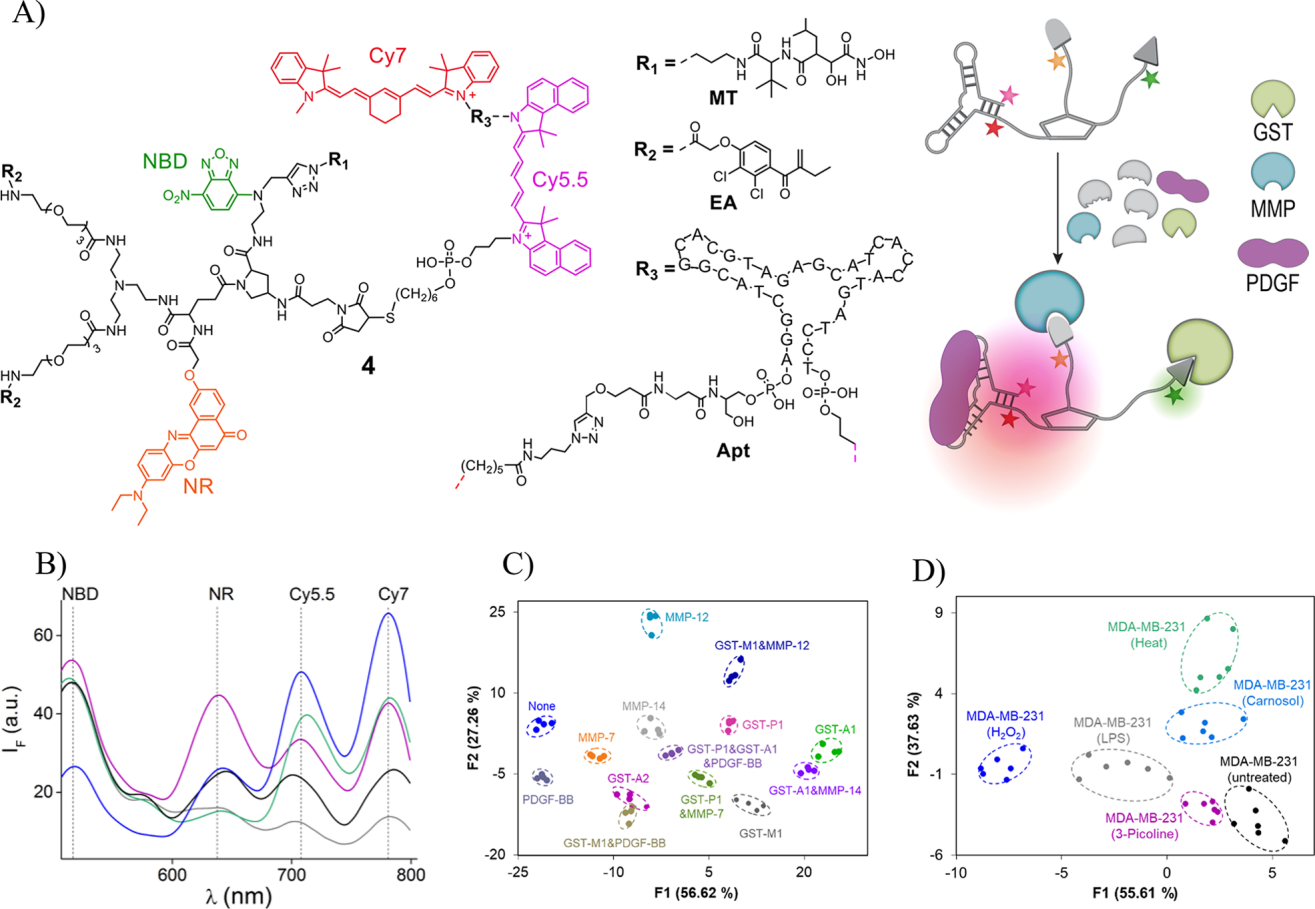
(A) Structure of ID-probe **4** (left) that can differentiate between specific populations of isoforms in biological mixtures and in living cells (right). (B) Representative emission patterns generated by **4** in the absence (black) and presence of GST-M-1 (magenta), MMP-12 (green), MMP-14 (blue), or PDGF-BB (gray). (C) LDA of the patterns generated by **4** in response to different isoform combinations in human urine. (D) LDA differentiation of nonengineered cells loaded with **4** after being subjected to heat, H_2_O_2_- induced oxidative stress, an inflammatory agent (LPS), or pharmaceuticals (picoline or carnosol). Adapted with permission from ref (3). Copyright 2017 Springer Nature.

Our understanding of various cellular functions and disease states largely depends on our ability to analyze protein subpopulations. However, the common analytical methods, such as western blotting or immunofluorescence, are less suitable for determining the composition of specific protein families in their native environment. By developing ID-probe **4** (Figure 4A, left), which can differentiate between isoforms of glutathione-S-transferases (GSTs), matrix metalloproteases (MMPs), and platelet-derived growth factors (PDGFs) in complex mixtures (Figure 4A, right), we demonstrated a potential means to address this problem.^3^ These protein groups were selected as analytes, because combinations of isoforms from these families have been detected in different diseases. The protein binders of **4** consist of a marimastat (MT), a bis-ethacrynic amide (bis-EA), and an anti-PDGF DNA aptamer (Apt), known to bind to different members of the MMP, GST, and PDGF isoform families, respectively. For the fluorescent reporters of **4,** we selected two solvatochromic dyes: nitrobenzoxadiazole (NBD), Nile red (NR), as well as cyanine 5.5 (Cy5.5) and cyanine 7 (Cy7). Incubating **4** with different isoforms leads to the generation of distinct emission fingerprints (Figure 4B), resulting from differential changes in the distance between the dyes and the polarity of the environment of NBD and NR. The selectivity of **4** toward GSTs, MMPs, and PDGFs was confirmed by its ability to discriminate between isoform combinations in complex mixtures, including human urine (Figure 4C).

To show that **4** can identify isoforms in living cells, different GSTs were expressed in HEK293T cells; then the engineered cells were loaded with the ID-probe. Recording the emission pattern generated from 35 individual cells revealed that similar fingerprints were generated only from those cells that expressed the same isoform. Moreover, **4** could classify individual, nonengineered living cells that were subjected to conditions such as heat, H_2_O_2_-induced oxidative stress, inflammatory agents, and pharmaceuticals, which change the composition of the isoform populations (Figure 4D). The implications of these experiments are tremendous. First, they showed for the first time, the feasibility of creating artificial “nose/tongue” devices that can operate in confined microscopic environments. Second, they indicated that unimolecular, pattern-generating systems can analyze intracellular states associated with diseases. Additional advantages of the ID-probe technology over arrays were also demonstrated. For example, we showed that ID-probe **4** can be easily recycled and used multiple times. In addition, **4** was used to construct a high-throughput screening (HTS) assay that can simultaneously detect small-molecule inhibitors of distinct proteins.

### 3.4 Information Protection and User Authentication at the Molecular Scale

An exciting direction in molecular logic and computing^32^ is to use fluorescent molecular probes to secure information.^42,43^ We realized that the small scale of ID-probes, together with their ability to generate distinct optical signatures, should make them superior to other molecular information protection systems intended to hide (steganography), encrypt (cryptography), or prevent access (password-protection) to information.

#### 3.4.1 Authorizing Multiple Chemical Passwords

The first molecular keypad lock, developed by Margulies and Shanzer,^44^ was based on a fluorescent molecular switch that “turns-on” only when specific chemical and optical inputs are introduced in the correct order. Although a variety of molecular keypad locks have been developed since then,^42,43^ they were generally designed to recognize specific inputs and produce only a few output signals. This prevented them from authorizing multiple passwords.

**Figure 5:**
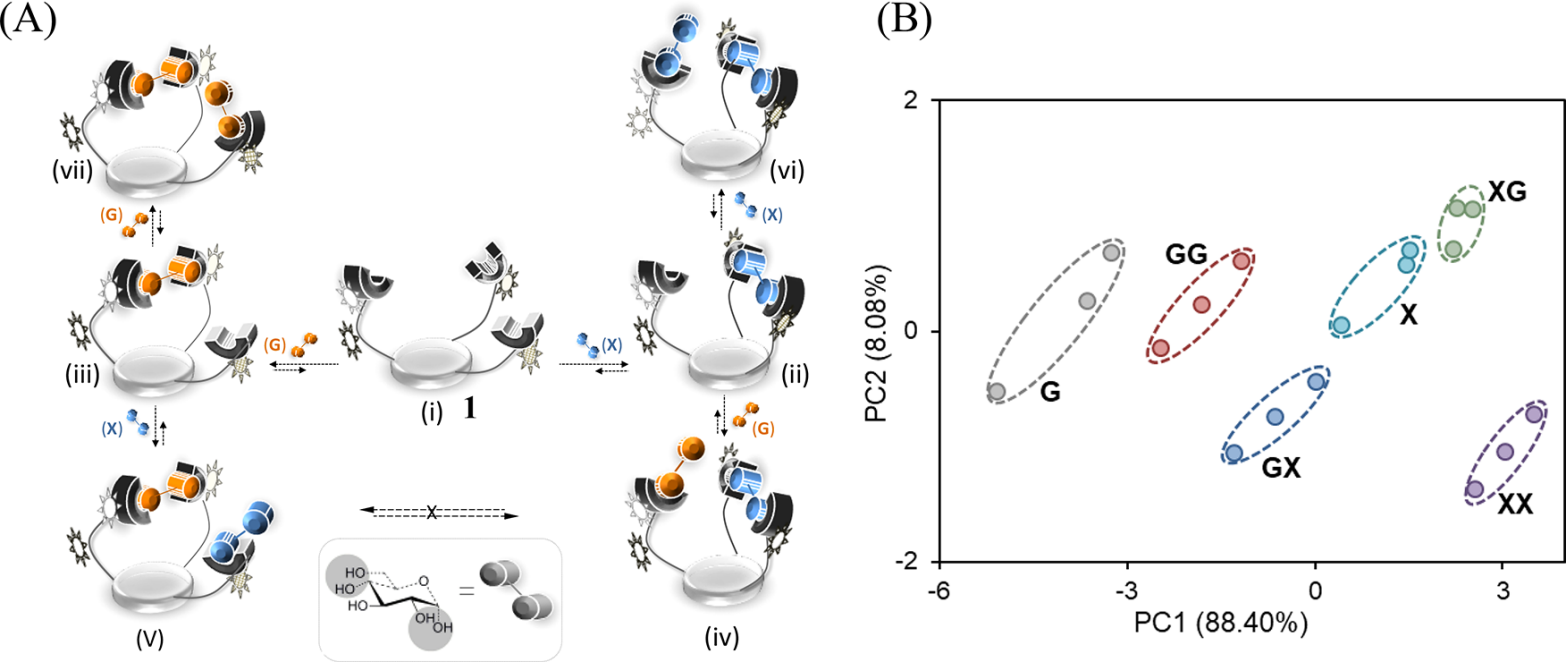
(A) Possible complexes that can be formed by adding d-glucose (G) and d-xylose (X) in different orders (iv vs v) or different concentrations (ii vs vi and iii vs vii). (B) PCA mapping of emission patterns generated by **1** in response to such code entries. Adapted from ref (36). Copyright 2013 American Chemical Society.

Our expectation that ID-probe **1** (Figure 2A) could operate as a far more advanced molecular keypad lock^36^ was based on the following assumptions: First, its ability to generate unique optical patterns for a wide range of saccharides should substantially increase the number of input keys. Second, because the emission pattern generated by **1** is affected by changes in input concentrations, it should be able to distinguish between password entries containing identical chemical inputs. Finally, we anticipated that the multivalent binding of this ID-probe to some saccharides would induce the formation of kinetically stable states, which should allow it to distinguish between input sequences. Figure 5A illustrates how these properties enabled **1** to authorize the six possible 1- and 2-digit code entries generated by adding two distinct saccharide inputs: d-glucose (G) and d-xylose (X) in different orders (iv vs v) or at different concentrations (ii vs vi and iii vs vii). In this scheme, very simple codes, namely, X, G, XX, GG, XG, and GX, can be distinguished. By introducing additional chemical inputs and analyzing the patterns generated in response to longer code entries, we showed that **1** can, in principle, authorize 81 different 4-digit combination codes.^36^

#### 3.4.2 Cryptography, Steganography, and Password Protection with an “Enigma-like” Molecular Machine

A key principle underlying the function of cryptographic (cipher) machines is the ability to convert a text message into nearly (pseudo) random letters. It occurred to us that the unpredictable fluorescence patterns that ID-probes generate could provide the means to encrypt and decrypt secret messages using randomly selected chemical inputs and a molecule-size cipher machine.^2^ One of the most famous cipher machines ever developed is the Enigma machine, which was used to encrypt messages during World War II. What significantly complicated cracking the enigma code was the enormous difficulty to reproduce the initial settings in enigma’s rotors. The need to set up the correct initial state of the device ensured that even if an enemy acquired an identical Enigma machine, decrypting messages would be extremely challenging.

**Figure 6:**
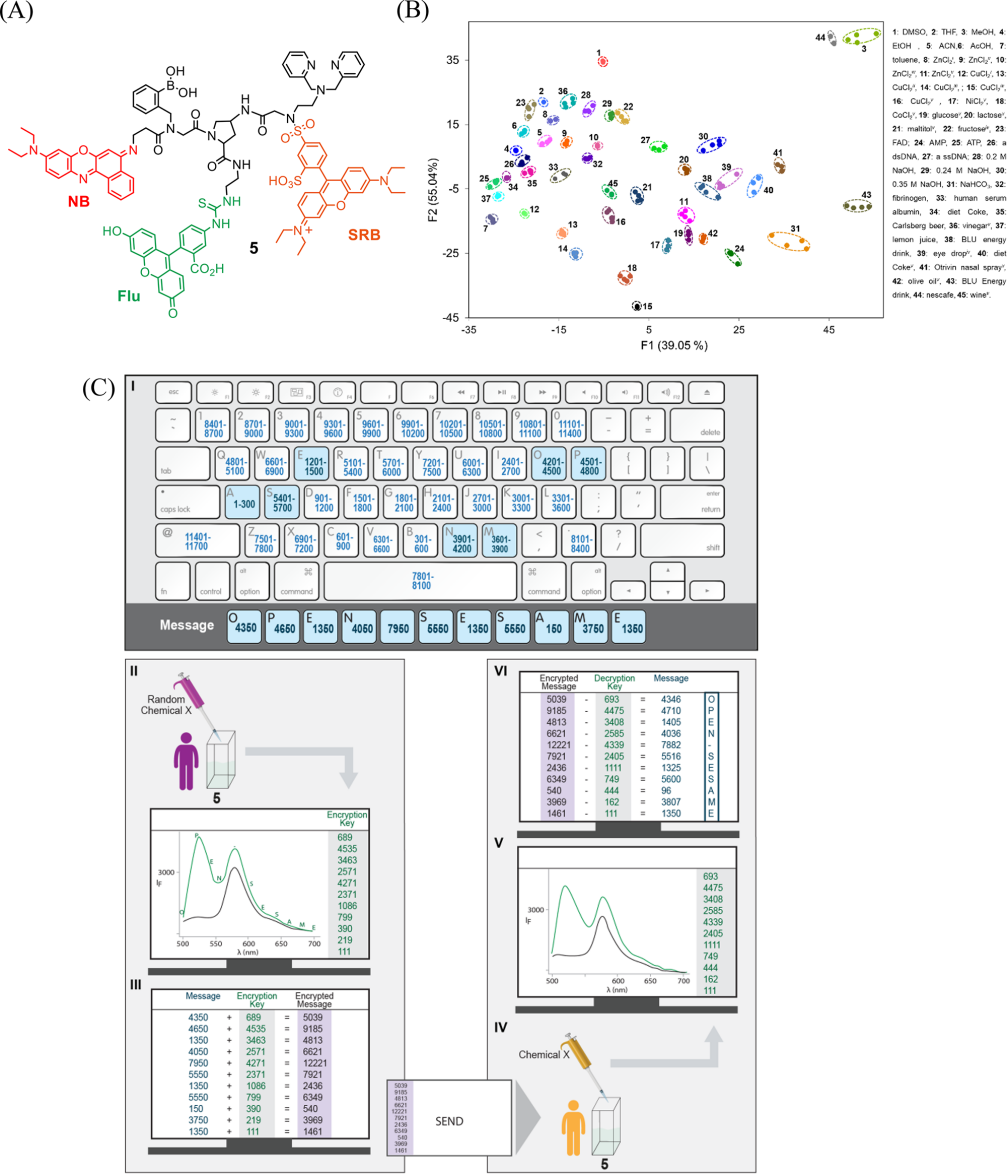
(A) Structure of ID-probe **5**. (B) LDA of 45 representative patterns generated by different analytes under diverse conditions. (C) Encrypting and decrypting a message with a molecule-size “enigma” machine (ID-probe **5**). Adapted with permission from ref (2). Copyright 2016 the authors. Published by Springer Nature under a Creative Commons Attribution 4.0 International License.

ID-probe **5** (Figure 6A)^2^ was created to generate a molecule-size cipher machine that, similar to Enigma, can generate pseudo random patterns that depend on its initial state. Unlike the previously discussed ID-probes, which were designed to respond to saccharides,^1,36^ Aβ aggregates,^35^ replicators,^41^ or proteins,^3^**5** was designed as a “universal sensor” that can generate unique patterns for a wide range of analytes. In addition to three fluorophores: fluorescein (Flu), SRB, and Nile blue (NB), the amino proline scaffold of **5** is appended with various recognition elements for binding distinct chemical species (Figure 6A). The boronic acid and dipicolylamine (DPA) groups, for example, can interact with various saccharides and metal ions, respectively, whereas thiourea and sulfonamide serve as additional metal ion-binding sites, anion receptors, and hydrogen-bonding motifs. The responsiveness of Flu to pH changes and the solvatochromism of NB further contributes to the ability of **5** to discriminate between various analytes, including commercial ingredients that can be obtained in grocery stores or pharmacies (Figure 6B).

Encrypting a message with **5** is fairly simple (Figure 6C). The sender merely needs to convert the letters to numbers using a public alphanumeric code (Figure 6C-I) and then measure the emission pattern generated by **5** after adding a randomly selected chemical (green line) (II). The intensity values, recorded every 20 nm, provide the encryption key. Adding the encryption key to the numeric message affords an encrypted message (cipher text) (III) that can be safely sent to the recipient. To decrypt the message, the recipient needs to generate the decryption key by setting up the correct initial state of the system (e.g., by using the appropriate solvent, sensor concentration, and detector gain) and measure the fluorescence spectrum of **5** in the presence of the same chemical input (IV → V). The original message is revealed by subtracting the decryption key from the cipher text (VI). Two additional layers of protection were reported in this work.^2^ One is password protection. We have shown that, with some chemical inputs, **5** can act as a molecular keypad lock. Hence, with such inputs, the enemy also needs to know the “chemical password” needed to generate the decryption key. The final layer of protection is steganography. We showed that, similar to secret inks, low quantities of the molecular cipher machine (**5**) can be concealed on paper and sent to recipients by regular mail. This not only complicates its detection—it also complicates its characterization, which would be needed if an enemy attempts to reproduce the molecular device. Overall, this work showed that cracking messages encrypted by ID-probes is almost impossible because they are protected by three different defense mechanisms: steganography, cryptography, and by entering a password, which are used to hide, encrypt, or prevent access to the information, respectively.

## 4 Self-Assembled ID-Probes

**Figure 7:**
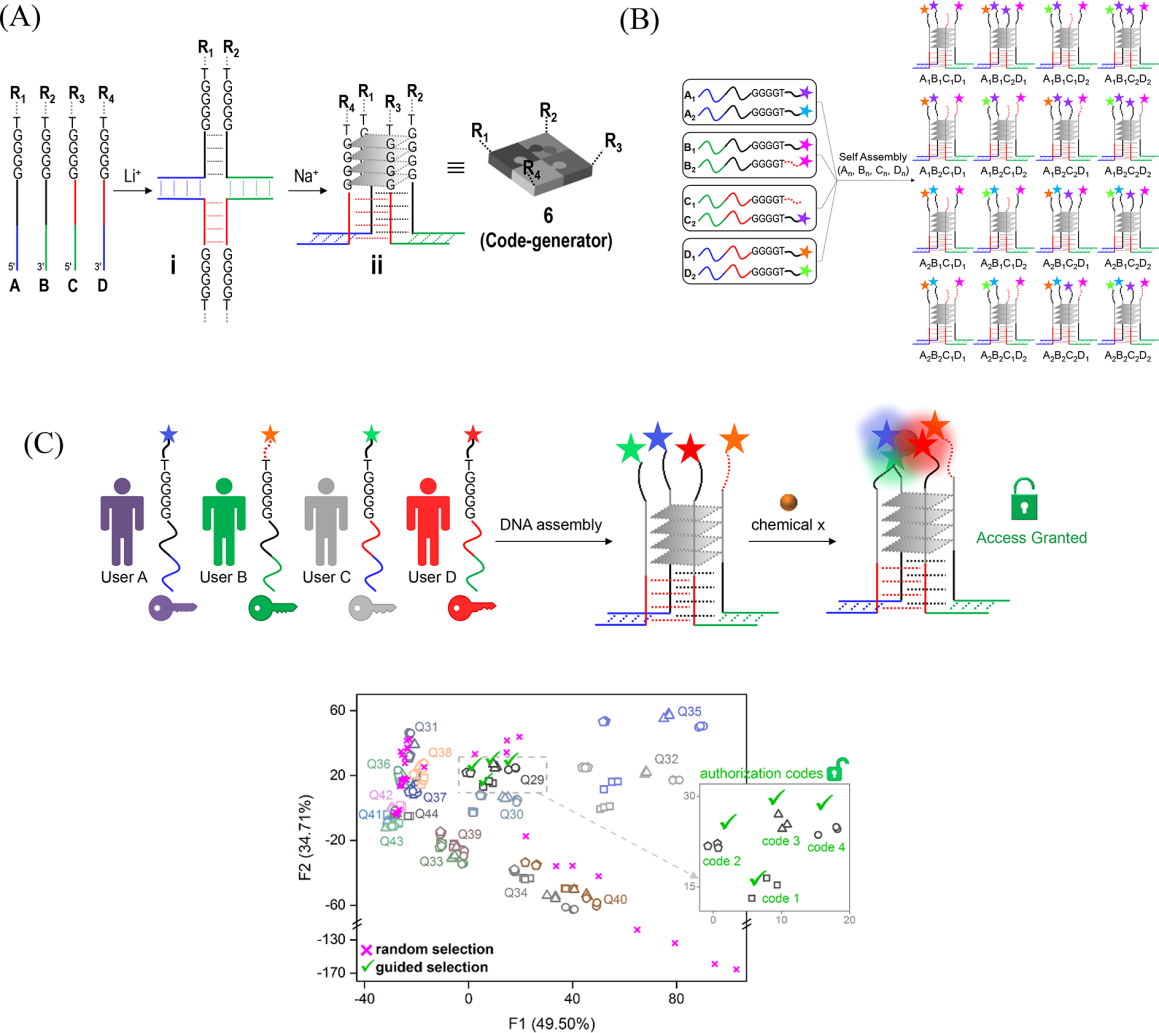
(A) Steps for creating a code generator (ID-probe **6**) based on an asymmetric and antiparallel G-quadruplex. (B) Schematic representation of the way libraries of code generators can be generated. (C) Top: Representation of a molecular secret sharing scheme. Bottom: An LDA map of the identification codes generated by various G-quadruplexes in response to distinct inputs. Adapted with permission from ref (34). Copyright 2019 John Wiley & Sons, Inc.

**Figure 8:**
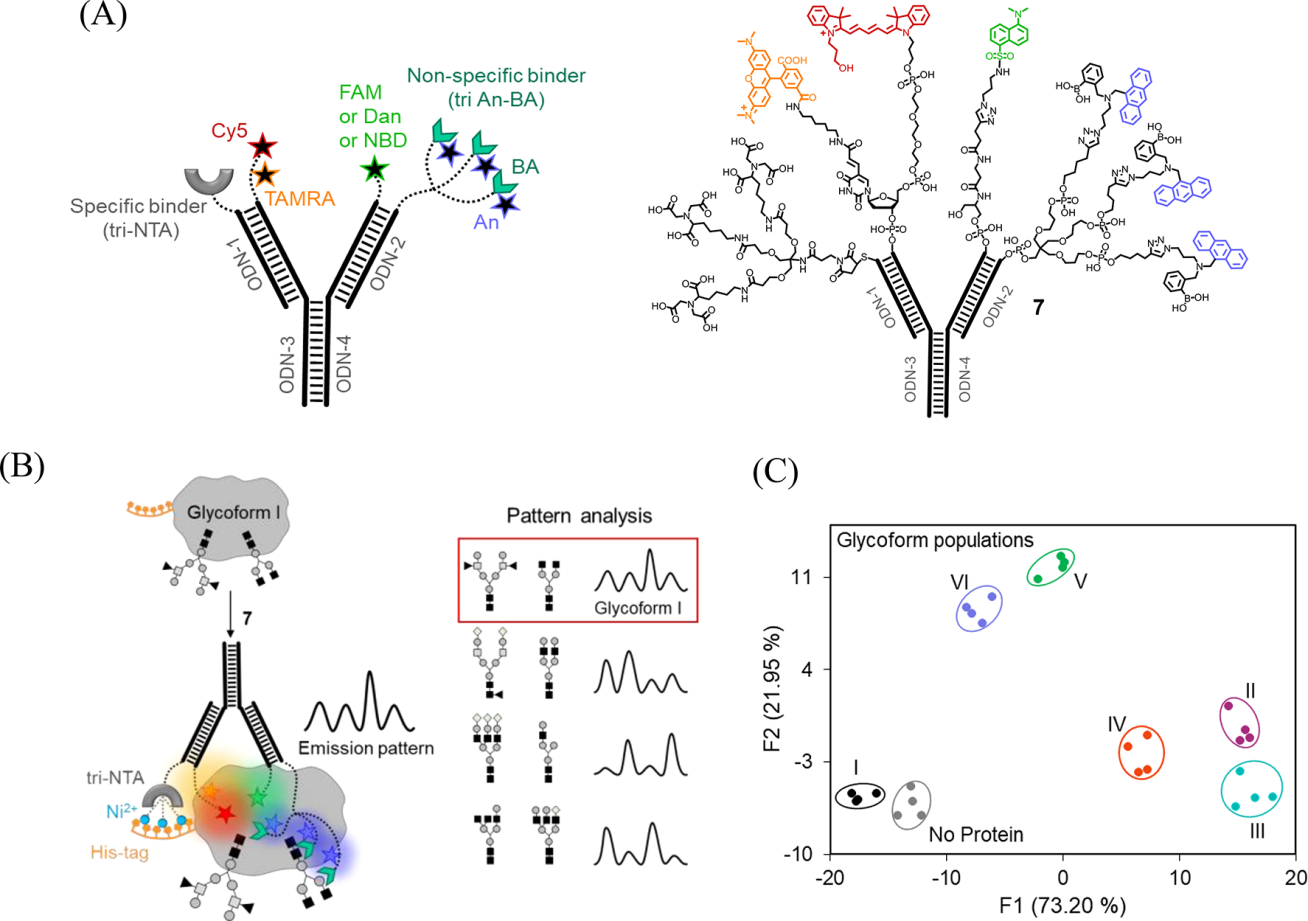
(A) Schematic presentation (left) and the structure (right) of the self-assembled ID-probe **7**. (B) Operating principles of **7**. (C) LDA of the emission patterns generated by **7** in response to different glycoform populations. Adapted from ref (4). Copyright 2020 American Chemical Society.

One limitation of the unimolecular ID-probes previously discussed (Figures 2–6) is the synthetic complexity of combining several fluorescent dyes and recognition elements within a single molecule. To address this issue, ID-probes based on self-assembled DNA scaffolds were created (Figures 7 and 8).^4,34^ The use of oligodeoxynucleotides (ODNs) to scaffold these probes endows them with inherent water solubility and provides a simple means to construct multiple different ID-probes with tailor-made molecular architectures.

### 4.1 Medication Detection and a Molecular Secret Sharing Scheme with ID-Probes Based on Intermolecular, Asymmetric, and Antiparallel DNA G-Quadruplexes

The self-assembled ID-probe **6** (Figure 7A)^34^ was generated from four modified ODNs; each contains a duplex-forming sequence, a quadruplex-forming guanosine repeat (GGGG), and recognition and signaling elements (R_1_, R_2_, R_3_, or R_4_). Quadruplex assembly takes place in two steps. First, a holliday junction is generated in the presence of lithium ions. Then, sodium ions are added that induce the formation of a G-quadruplex. Our design is inspired by previous studies showing the creation of synthetic receptors and sensors from symmetric, intermolecular, and parallel DNA G-quadruplexes.^45^ A unique feature of our design, however, is that it provides the means to create an asymmetric and antiparallel G-quadruplex structure. In this way, libraries of ID-probes that bear distinct synthetic receptors and fluorescent reporters (R_1_-R_4_) can be readily created (Figure 7B). Similar to the first ID-probe **1**(1,36) (Figure 2A), the self-assembled ID-probe **6**(34) was first applied to discriminate between medications and drugs of abuse and later to authorize users. Using **6** instead of **1** had two main advantages: First, in terms of drug identification, the ability to generate a wide range of modified G-quadruplexes (Figure 7B) enabled us to test the response of various ID-probes and select the ones that provide the best differentiation. Second, in terms of user authorization, we have shown that it is possible, for the first time, to apply a secret sharing scheme at the molecular level (Figure 7C). Specifically, we have shown that because the ODNs constituting **6** can be distributed among several participants, an authorization code can only be generated when all the participants are present (Figure 7C, top).

### 4.2 Glycoform Differentiation Using a DNA-Based Protein Surface Sensor

Fast and efficient methods for analyzing protein glycosylation could improve our ability to diagnose diseases or control the quality of therapeutic glycoproteins. Although mass spectrometry and lectin arrays are commonly used in glycoprotein analysis, these methods are generally not high throughput. The self-assembled ID-probe **7** (Figure 8A)^4^ was designed to straightforwardly differentiate between intact glycoproteins by taking a single fluorescent measurement (Figure 8B). The structure of **7**(4) consists of four modified ODNs that self-assemble into a Y-shaped scaffold resembling antibodies (Figure 8A). ODN-1 is appended with a highly specific tri-NTA binder of a hexa-histidine tag (His-tag),^46,47^ ODN-2 is linked to a nonspecific, tripodal glycan binder that was generated from the well-known anthracene-boronic acid (An-BA) fluorescent probe,^10^ whereas ODN-3 and ODN-4 bear the fluorescent reporters, namely, Cy3, Cy5 and FAM, Dan, or NBD.

Based on our previous studies on protein surface sensors^29,46,48,49^ and other synthetic agents that interact with protein surfaces,^50^ we expected that the strong interaction between the sensor and the glycoprotein’s His-tag would promote the binding of the nonspecific glycan binder and other fluorescent groups to its surface. This should change the chemical environment of the dyes and the distance between them, which would lead to the generation of a unique emission fingerprint for each glycosylation state (Figure 8B). The ability of **7** to straightforwardly analyze the glycosylation states of a His-tagged human chorionic gonadotropin (hCG) (Figure 8C), a glycoprotein given to women as part of assisted reproductive technology, demonstrated the analytical power of this approach. In addition, it showed the feasibility of integrating a wealth of supramolecular receptors and sensors into higher-order molecular analytical devices with advanced properties. For example, the facile device integration enabled us to incorporate a trinitrilotriacetic acid (tri-NTA)-Ni^2+^ complex into the sensor,^46,47^ which endows it with selectivity toward a His-tag. Furthermore, it enabled modifying the DNA scaffold with the An-BA probe, which is one of the most interesting and well-studied “turn-on” fluorescent probes in supramolecular chemistry. This provided the means to use the unique photophysical properties of An-BA^10^ to improve glycoform differentiation. Specifically, the integration of An-BA with solvatochromic probes (e.g., Dan or NBD) and two FRET acceptors (i.e., TAMRA and Cy5) afforded an exceptional fluorescent molecular sensor (ID-probe **7**) whose fluorescence is affected by changes in five different photophysical processes: PET, ICT, FRET, internal conversion, and excimer emission.

## 5 Summary and Outlook

With this Account, we hope to attract attention to the emergence of a new class of fluorescent molecular sensors, termed ID-probes, which integrate the properties of unimolecular fluorescent sensors and cross-reactive sensor arrays. On the one hand, such probes can discriminate between a wide range of analyte combinations, akin to pattern-generating arrays. On the other hand, similar to small-molecule-based probes, they can analyze small-volume samples, track changes that occur in a single solution, and operate in the microscopic world, where macroscopic arrays cannot access. By modifying a *cis*-amino proline scaffold with different dyes and synthetic receptors, we have demonstrated the possibility of tailoring such probes (ID-probes **1**–**5**) to different applications, such as pharmaceutical quality assurance, medical diagnosis, analytical proteomics, and information protection. Despite this progress, making this technology relevant to real-world applications would require novel methods to optimize the ID-probes’ performance, for example, by enhancing their differentiation ability or by simplifying their preparation. One way to address these problems is by generating self-assembled ID-probes, such as probes **6** and **7**. Although this approach offers a simple means to generate multiple ID-probes and select the best ones for a desired application, it also has some limitations. For example, the DNA scaffold may dissociate under certain conditions; hence, applying self-assembled ID-probes to sense analyte combinations in an intracellular environment could be challenging. Moreover, because small alterations in the concentration of the ODNs constituting the self-assembled ID-probes can change their fluorescence patterns, achieving batch-to-batch reproducibility with such probes requires extra caution when manufacturing them. We hope this perspective will inspire scientists to develop additional means to further enhance the properties of ID-probes and consequently, expand the ways and forms in which this emerging class of sensors could be utilized.
